# An operational framework for wildlife health in the One Health approach

**DOI:** 10.1016/j.onehlt.2024.100922

**Published:** 2024-10-24

**Authors:** C. Goulet, M. de Garine-Wichatitsky, P. Chardonnet, L.-M. de Klerk, R. Kock, S. Muset, R. Suu-Ire, A. Caron

**Affiliations:** a Colibri consulting, Maputo, Mozambique; b ASTRE, University of Montpellier, CIRAD, INRAE, MUSE, Montpellier, France; c International Union for Conservation of Nature (IUCN), SSC Antelope Specialist Group, Gland, Switzerland; d International Union for Conservation of Nature (IUCN), SSC Wildlife Health Specialits Group, Switzerland; e Department of Agriculture, Land Reform and Rural Development (DALRRD), State Veterinary Office & Laboratory, Kruger National Park, Skukuza, South Africa; f Royal Veterinary College (RVC), London, United Kingdom; g World Organisation for Animal Health, Paris, France; h School of Veterinary Medicine, University of Ghana, Accra, Ghana; i International Livestock Research Institute (ILRI), Nairobi, Kenya

**Keywords:** Wildlife, Wildlife health, One Health

## Abstract

Wildlife is an essential component of biodiversity and provides people with multiple social and economic benefits. However, a resurgence of epidemics over the past two decades has highlighted wildlife's role as a potential source of dangerous pathogens for humans and livestock, with devastating consequences worldwide. Simultaneously, numerous reports have indicated that wildlife populations are declining at an alarming rate due to human and livestock pathogens, predation, and competition. An integrated approach to managing wildlife, human, and domestic animal health is therefore clearly needed. Yet this integration often fails to materialize due to a lack of wildlife health standards and know-how. Here, we present an operational framework that follows a step-by-step approach: i) a holistic definition of human health is adapted to the context of other-than-human animals, including wildlife; then, ii) different categories of wildlife living within a landscape or a country are defined based on the management systems under which they live. For each wildlife category, the type (natural vs. anthropogenic) of habitat, the nature of the interface of wildlife with humans and/or livestock, and the level of sanitary control are defined; and finally, iii) the holistic definition of wildlife health is considered in relation to each wildlife category to define health challenges and the domains of expertise required to address them. This framework can assist national and international agencies, including veterinary and wildlife authorities and policy makers, in defining wildlife health priorities, responsibilities, policies and capacity building strategies. The extensive interdisciplinary collaboration needed to manage the many different aspects of wildlife health calls for a more integrated One Health approach.

## 1 Introduction

The health of humans and of other-than-human animals is deeply interconnected, extending beyond the sharing of pathogens and diseases. The many material and non-material relationships that humans have with the animals sharing their environment have contributed to the development of human societies [1]. In the process, the health of both domestic and wild animals has been affected by human practices and impacts [2].

The recent emergence of infectious diseases in humans that are related to domestic or wild animals, i.e., zoonoses, has focused public attention on the potential health threats associated with the connection between humans and animals. Zoonotic pathogens can be transmitted between different host species through spillover events occurring at domestic animal/human or wildlife/human interfaces [3]. In the past twenty years, several epidemic events caused by emerging pathogens originating from wild animals have shaken human societies [4]. Examples include SARS-COV in 2003 [5], Ebola disease (in West Africa) in 2014 [6] and more recently the COVID-19 pandemic in 2020 [7]. However, this focus on zoonoses should not hide the numerous threats to wildlife populations caused by humans encroaching into natural habitats and making use of wildlife. The resulting increased contact between wildlife, domestic animals and humans raises not only the risk of pathogen spillover to wild animals, but also of many wild populations becoming extinct through direct competition and exploitation [8,9]. The management of the health of other-than-human animals, especially wildlife, is therefore paramount not only for human health, but also for their own.

The terms “wildlife” and “wild animals” are often used interchangeably, the former being the most common. In its Terrestrial Code, the World Organisation for Animal Health (WOAH) defines “wildlife” as “feral animals, captive wild animals and wild animals”, “wild animal” as an “animal that has a phenotype unaffected by human selection and lives independently without requiring human supervision or control”, and “animal” as a “mammal, reptile, bird or bee” [10]. The Terrestrial Code provides global animal health standards for countries to set up their legislation concerning animal production, trade, health, surveillance, and control. In this article, we will use the term “wildlife” and “wild animals” following the WOAH definitions, excluding therefore wild plants and invertebrates.

The One Health concept offers a path towards a holistic and muti-dimensional management of wildlife health through a systemic, transdisciplinary, cross-sectoral, and inclusive approach to health [[11], [12], [13], [14]]. As wildlife are part of the ecology of multi-host pathogens, the implementation of wildlife health surveillance systems using a One Health approach has been promoted to protect the health of human and livestock populations against spillover from wildlife [15]. Since the modern definition of One Health was established in the 2000s (e.g., [16]), the focus of One Health has been dominated by the human and livestock health sectors, with a major anthropocentric perspective. For example, the initial tripartite One Health agreement between the World Health Organisation (WHO), WOAH and the Food and Agriculture Organisation (FAO) paid little attention to either environmental health or to wildlife health, save for the transmission of infectious diseases to domestic animals and humans [17]. Calls for a return to the original vision of One Health [18,19] were answered in 2022 with a new definition of the One Health concept by the One Health High-Level Expert Panel (OHHLEP) [20]. This followed the integration of the United Nations Environment Programme (UNEP) as a new quadripartite partner. The strategic orientations of the joint plan of action issued in 2022 by the quadripartite emphasize the desire to move away from an anthropocentric perspective of the One Health concept [21]. Many initiatives are currently being developed to better integrate the environmental component and wildlife health into One Health approaches (e.g., [22]). However, thus far no consensus has been reached regarding the operational framework to be used to achieve this integration based on international standards. Consequently, countries are left to their own devices when it comes to putting in place wildlife health management options. In contexts where there is a lack of expertise and resources, this results in poor or even no wildlife health management.

Wildlife populations around the world inhabit diverse ecosystems and are subject to various management regimes, which result in different relationships with livestock and humans. Humans use and interact with wildlife in many different ways. Different approaches to managing wildlife health are therefore required, adding to the complexity of defining regulatory frameworks. For wildlife health to be integrated into the implementation of the One Health concept, and for countries to define how to manage wildlife health, one needs to understand what wildlife health means and encompasses.

This article proposes an operational framework and a step-by-step approach to help national and international agencies, including wildlife authorities and policy makers, to effectively integrate wildlife health into their national policies and capacity building strategies. The framework considers the diverse categories of wildlife and identifies the appropriate range of disciplines and stakeholders required to tailor wildlife health management to wildlife categories.

## 2 Why does adopting an operational framework for wildlife health matter?

Wildlife is not only a source of pathogens, but also an essential part of biodiversity, which is currently undergoing a human-induced crisis [23]. The ecological value of wildlife has been disregarded for centuries, especially in Western societies [2]. Yet wildlife plays a critical role in biodiversity by contributing to ecological functions at the basis of healthy ecosystems, including agrosystems [24]. According to the Intergovernmental Science-Policy Platform on Biodiversity and Ecosystem Services (IPBES), wildlife, as part of nature, contributes to people through material and non-material Nature Contributions to People (NCP) [25]. For these reasons, healthy wildlife should be considered as a public good that is needed for both biodiversity conservation and human well-being [26]. The white-nose syndrome affecting bat populations in North America illustrates the role of wildlife in ecosystems. Caused by a fungus (*Pseudogymnoascus destructans*), this deadly disease is currently decimating bat populations in North America [27], threatening the multiple NCP that bats provide. These services are crucial for maintaining ecological functions and supporting agricultural systems. The decline in bat populations due to white-nose syndrome is estimated to be causing annually a $3 billion loss to the agriculture sector of the United States of America (USA) [17,28,29]. It is now widely agreed that wildlife health must be better managed, not only for its intrinsic value, but also for its socio-ecological function and its potential impact on human and domestic animal health. However, managing wildlife health can mean very different things depending on the context in which wildlife populations live. This point is illustrated by two examples, the American mink (*Neogale vison*) and the African buffalo (*Syncerus caffer*) (Box 1, Box 2). In free-roaming populations, the relationship with endemic pathogens in a natural setting is considered part of the ecological functioning of ecosystems, just like competition for resources between populations or predation by one species on another [30]. Unlike farmed wildlife, there is no need for human intervention to eradicate diseases affecting viable free-roaming wildlife populations. Health interventions may be justified in free-roaming wildlife only when populations are small or endangered due to habitat loss, harvesting or other anthropogenic impacts, and they risk extinction. However, even in such cases, the best option is to eliminate potential threats by controlling diseases in domestic animals and humans before they spread to wildlife. For example, eradicating epidemic diseases like peste des petits ruminants (PPR) in domestic goats and sheep can reduce the risk of weather-related pathogen expression and the threat posed to wild antelope populations [8,31].

Box 1 **Managing the health of American minks.**

American mink (*Neogale vison*) populations occur under different conditions within the same country. By applying the first steps of our framework to Canada as an example, we can define four categories of mink populations. In certain parts of Canada, American mink populations roam freely in their natural habitats, where they find food and refuge. Other mink populations are bred for fur under intensive farming conditions. They are caged, fed, and their reproduction is controlled. Over generations, they have been selectively bred for the main purpose of maximizing their fur production. From time to time, some farmed minks have escaped from their farms and have produced “feral” mink populations. These behave like free-roaming mink, but they are genetically different after generations of human-driven selection. Finally, some mink individuals are kept in zoos to show visitors examples of Canadian indigenous species. Living in enclosures that replicate their natural habitat, they are fed and their reproduction is controlled.

Each of these mink populations experiences and poses different health risks depending on the contact they have with other animals, including humans. According to the bio-statistical component of health (see Table 1), free-roaming mink populations are wild, and as such are exposed to natural pathogens which impact the population. However, these natural pathogens do not require any health management as they belong to natural processes that regulate the free-roaming mink population. On the other hand, free-roaming mink numbers have been declining in Canada. One hypothesis to explain this is that breeding with feral mink populations (i.e., animals originating from escaped farmed individuals) has led to the transmission of a highly pathogenic parvovirus causing Aleutian disease, which can impact the fitness of the free-roaming mink populations [32,33]. This health challenge to free-roaming mink populations requires some management intervention (e.g., by controlling feral mink populations). The feral mink population is considered to be an invasive population threatening the free-roaming population. This threat can materialize through introgression of farmed-selected traits, but also through the transmission of pathogens, such as the SARS-COV 2 coronavirus [34]. The health of farmed mink populations is closely monitored, not only to ensure the maintenance of fur production, but also to protect farm workers from any zoonotic transmission (e.g., SARS-COV 2) [35]. In zoos, the health of individual minks is also monitored to keep animals in good condition for their own well-being and for the benefit of zoo visitors.

The prism of the holistic component of health (see Table 1) provides different perspectives for each mink population category in Canada. Free-roaming mink populations legitimately deserve to live a decent life, they contribute to functional and healthy ecosystems, and they should benefit from sound conservation programmes. Civil society movements and scientific communities are promoting animal welfare and animal rights as important pillars of mink health for populations in farm and zoo environments [36]. Meanwhile, due to the threat they pose to free-roaming populations, feral mink populations should be eradicated using means respecting animal welfare standards. This management of feral mink populations aims to preserve the conservation of free-roaming minks in their natural habitat (i.e., part of holistic component). In Europe, the same situation threatens the survival of the European mink (*Mustela lutreola*) [37]. Such feral populations should be eradicated to prevent an impact on free-roaming populations, using means respecting animal welfare standards. This management of the feral mink population aims to preserve the conservation of free-roaming minks in their natural habitat (i.e., part of holistic component).

Therefore, the four categories of mink populations in Canada require different health management programmes adapted to the habitats and the type of management systems under which the minks live. These factors define the level/frequency/intensity of mink/human interactions and the level of sanitary control required for each mink population.

Alt-text: Box 1

Box 2 **Managing the health of African buffalo.**

In South Africa, there are several categories of African buffalo (*Syncerus caffer*) populations. Firstly, in many national parks, buffalo live under natural conditions in the species' natural range [38]. They are exposed to predation and endemic diseases (e.g., foot-and-mouth disease (FMD) SAT serotypes and theileriosis caused by *Theileria parva*), and reproduce freely. Over the past 30 years, South Africa has seen the emergence of a booming wildlife sector and diverse buffalo production systems. These range from extensive ranching on large game farms with conditions closely resembling natural ones to intensive farming where buffalo are kept in feedlots or small enclosures with controlled feeding and breeding (e.g., artificial insemination) [39]. These production systems produce buffalo for trophy hunting, leisure or translocation into (mostly private) protected areas. Some individuals are also kept in zoos for visitors to view. Hunted and butchered buffalo, mainly from ranched populations, represent a separate category as they are transformed into meat consumed by humans, thereby creating a specific interaction at the buffalo/human interface.

As in the mink example (Box 1), each of these buffalo populations experiences and poses different health risks depending on the contact they have with other animals, including humans. According to the bio-statistical component of health (see Table 1), free-roaming buffalo populations (e.g., in Kruger National Park) live with and adapt to endemic diseases with which they have co-evolved. The interaction between these pathogens and buffalo should be considered part of natural processes. However, the picture becomes more complex since exotic pathogens of colonial origin have spilled over from cattle to free-roaming buffalo populations (e.g., bovine tuberculosis - bTB) [40]. These exotic diseases should be managed, not only to preserve buffalo health, but also to prevent spillover into other wildlife populations (e.g., lion, *Panthera leo*), and spillback into livestock and potentially into human populations at wildlife/livestock/human interfaces. Three-quarters of South African buffalo live on ranches and farms, many of which are located outside their historical range. Often adjacent to livestock populations, these buffalo populations are kept under strict sanitary surveillance, and most of them are “disease-free”, meaning free from FMD, bTB, theileriosis, brucellosis and Rift Valley fever related pathogens. The numerous movements of these individuals between farms and to protected areas or elsewhere are under strict sanitary control as well. As buffalo meat is one of the outputs of these buffalo production systems, it must undergo a specific veterinary public health inspection before entering the food chain. In zoos, the health of individual buffalo is also monitored to ensure that animals remain in good condition in a context of indirect and/or direct contact with other species and with zoo visitors.

If the holistic component of health (see Table 1) is now taken into consideration, free-roaming buffalo populations deserve the right to live a decent life, with healthy populations which can contribute to the future of the species and healthy ecosystems. Animal welfare considerations come into play for ranched and farmed buffalo. However, these differ depending on whether the buffalo are raised under close-to-natural conditions (as in extensive farming systems) or under close-to-livestock farming conditions (as in more intensive farming systems). Welfare aspects also should be considered during the hunting and slaughtering of individual buffalo for game meat. When considering the holistic component of health, the social and nutritional benefits of meat production (e.g., making healthy game meat available) also need to be considered. Finally, there are specific animal welfare considerations for zoo animals, including replicating as much as possible the settings of their natural environment within a closed environment (i.e., enrichment) and respecting the animals' social needs (e.g., living in a herd), which is often challenging in zoos.

Given the existence of multiple categories of buffalo populations, the South African government has developed specific regulations for certain wildlife categories (e.g., farmed/ranched buffalo [39,41]). However, the thin dividing line between these different categories, in particular ranched/farmed and free-roaming buffalo populations, poses risks for the commodification of the buffalo resource and threatens the conservation of free-roaming buffalo populations in natural areas [42].

Alt-text: Box 2

In farming and ranching situations, wild populations are managed by humans, who control their feeding, reproduction, and sanitary status. The aim of the production system is to produce goods, such as live animals, meat, and fur. The control of pathogens and diseases is necessary for the population to remain productive and to protect the health of farm workers and other humans from the spillover of zoonoses. The populations' living conditions resemble that of many livestock production systems, and include movement restrictions.

The examples presented in Box 1, Box 2 illustrate how wildlife populations of the same species living in contrasting situations may require drastically different health management strategies. However, many responsible government entities seem to lack specific strategies for dealing with this heterogeneity of situations for wildlife populations. The quasi absence of international standards for wildlife health (see recent publication [43]), does not facilitate the adoption of different strategies for the health of these populations [44]. Adopting a sensible One Health approach would lead to the definition of the different contexts in which wildlife populations live, and the legal and sectoral frameworks under which they fall. This would help managing agencies and policy makers to better define their wildlife veterinary public health and conservation management strategies, including field interventions, training/capacity building needs, and policy revisions.

In the following sections, we will present a step-by-step approach for institutions or countries aiming to develop an operational framework for wildlife health. In the first step, a comprehensive definition of health will be adapted to the context of other-than-human animals, including wildlife. In the second step, different categories of wildlife will be defined based on the management systems under which they live. For each wildlife category, the type of habitat (natural vs. anthropogenic), interface with humans and/or livestock, and sanitary control will need to be identified. In the final step, the holistic definition of wildlife health is considered in relation to each wildlife category to define health challenges and the domains of expertise required to address them. Once this has been achieved, all stakeholders involved should have a clear and shared understanding of what wildlife health entails in their specific context. This will make it possible to implement clear legislative guidelines and virtuous governance of wildlife health.

## 3 A holistic approach to health

Hanisch et al. [45] expanded on the WHO's definition of human health and applied it to wildlife health. WHO defines human health as having two components. The first component is rooted in bio-statistical theory and refers to sanitary objectives, including the presence or absence of diseases that impact – or not – a human individual or population. It covers domains such as medicine (individual level), epidemiology, and public health (population level). The second component is rooted in holistic theory and refers to health objectives associated with mental health and well-being. This second component relies on domains of expertise such as social and psychology sciences. This dichotomy is anchored in the WHO's definition of human health and new approaches to health described by some conservation experts [46]. Here we use this definition of human health and apply it to the health of other-than-human animals (Table 1). Table 1 illustrates how health objectives may differ according to the categories of living beings targeted, and highlights that the domains of expertise needed to achieve these health objectives vary.

**Table 1:** The different components of human and animal health and their definitions/attributes, adapted from Hanisch et al., 2012. “Level of health intervention” refers to the main level of health intervention on the health compartment, secondary levels are indicated within brackets. “Expertise” indicates some examples of scientific disciplines needed for each component of health. For both components (i.e., bio-statistical & holistic), as defined in Hanisch et al. 2012, the objectives of health interventions are given in the cells for each line.

	Level of health intervention	Bio-statistical component	Expertise (Ex.)	Holistic component	Expertise (Ex.)
Human Health	Individual Population	Absence of disease	Medicine Public Health	Mental Health Well-being	Social & Psycho. Sciences
Livestock Health	(Individual) Population (Community)	Absence of disease	Veterinary Sc. Epidemiology	Welfare Economic production	Animal welfare Economics
Wildlife Health	(Individual) Population Community	Natural disease dynamics	Disease Ecology & evolutionary biology	Viable population & community	Animal Welfare Ecology Conservation and biodiversity Sc. Evolutionary biology

In the context of human health, the objective within a bio-statistical perspective is to eliminate all diseases that lead to mortality and morbidity in human populations. Applied to domestic animals, the objective is similar: the goal of veterinary services is to eradicate or mitigate infectious and other diseases in animals used for human consumption, agricultural work, for recreational purposes or any other types of uses. There are two reasons: productivism (optimize the production and/or use of domestic animals) and sanitary reasons (manage zoonoses that can spillover from domestic animals to humans). Veterinary sciences and epidemiology are disciplines that are well adapted to support the goals of domestic animal health. If we consider initially free-roaming wildlife in its natural environment, applying the bio-statistical perspective to wildlife health does not mean targeting an absence of diseases. Free-roaming wildlife in their natural environment are in interaction with many other species in complex ecological networks, including pathogens (e.g., a free-roaming American mink population in forested habitat close to a river). In this context, the objective of wildlife health management (e.g., for a national authority or a conservationist) is to maintain natural disease dynamics allowing wildlife populations to be regulated by pathogens. In this situation, a thorough understanding of disease ecology and wildlife-related sciences is required (e.g., behavioural ecology).

For human health, the holistic theory defines health objectives related to individual mental health and well-being, and incorporates social and psychology sciences to support the achievement of these objectives. When applied to livestock health, the holistic theory covers two objectives. Firstly, animal welfare and animal rights movements recently have been putting increasing pressure on livestock production systems to improve animal well-being. Since 2013, the WOAH Performance Veterinary Services Pathway tool has included this criterion as a new component to be assessed in all animal sectors, domesticated and wild [47]. Secondly, when dealing with livestock production systems, the “healthiness” of the production system could also be included as a holistic component. This would ensure that the productive and economic performance of the production system is relevant. Achieving these two objectives will require expertise in areas such as animal welfare, livestock management, and economics. The holistic theory applied to free-roaming wildlife in its natural habitat should refer to the healthiness of a wild population, defined by its long-term viability and resilience to shocks (e.g., endemic disease outbreak) [46] in the absence of any human management. This relates to the field of biodiversity conservation. Animal welfare and animal rights issues at the population level can become a concern when specific management interventions, such as culling/slaughtering or translocation, are used on wildlife populations (e.g., culling, [48]), or when anthropogenic activities directly impact wildlife habitats (e.g., pollution of ecosystems by intensive agriculture). We will return to this holistic definition of health in the third step below.

## 4 Defining wildlife categories

Within a given context (e.g., a country or a landscape), our mink and buffalo examples (Box 1, Box 2) showcase that for a given wildlife species, different populations living under different management systems require different health management strategies. To define suitable health management strategies for wildlife populations, managers and decision-makers therefore need to identify the different wildlife populations living under different management systems. To explore the diversity of wildlife population management systems, a list of wildlife categories is presented in Table 2. It does not pretend to be exhaustive as categories are only subjective representations and depend on social, economic, and cultural perceptions, and should be defined according to context. For example, one could also consider wildlife populations subjected to research experiments.

**Table 2:** Non-exhaustive list of wildlife categories based on their level of management and use by humans. The column “Management system” describes the management system under which each category of wildlife lives. This management system defines the level of interface with humans, and thus potential risk factors for pathogen transmission at the human-domestic-wildlife interface and stressors to wildlife populations (e.g., habitat encroachment, hunting).

Categories	Brief description	Habitat	Management system	Interface / humans	Sanitary control
Free-roaming wildlife in its natural habitat	Wild animals living naturally, adapted to their habitat and landscape	Natural and anthropogenic habitats	None or protected area management	None to low in natural habitats	None to very low
Feral animals	Domestic animals free-ranging out of human control	Natural to anthropogenic habitats	None or population control when listed as pests	None to Medium	Low to Medium
Ranched wildlife	Wild animals living in fenced or restricted land management systems	Extensive land use but highly modified	Medium with feeding, reproduction, movement, health at population level	Medium	Medium to high
Farmed wildlife	Wildlife species kept as domestic production animals	Farm	Intensive production system	High	Medium to High
Harvested Wildlife (e.g., bushmeat)	Wild animals living in their natural habitat hunted/captured for subsistence or trade purposes	Natural, trade systems	Subsistence hunting to harvesting systems for legal and illegal commercial purposes	High (butchering, handling)	Low to High
Sport/ tourism hunting wildlife	Wild animals living in natural or ranched habitats hunted for leisure purposes	Natural to extensive controlled habitats	Leisure hunting	High (butchering, handling)	High
Pet wildlife	Wild animals captured or bred in captivity and kept as pets	House, trade system	Member of human familial social units	High	High
Peri-domestic wildlife	Wildlife populations living in human domains/anthropogenic habitats	Peri-domestic	None or population control when listed as pests	Medium to high	Low to High
Zoo and captive wildlife	Wild animals kept in public and private zoological collections	Zoo, human domain	High with control of feeding, reproduction, movement, health at individual level	Medium	High

This wildlife categorization process involves identifying the following elements: i) the habitat in which a wildlife population lives and the degree to which anthropogenic activities impact this habitat; ii) the type of management system applied to this wildlife population; iii) the level of wildlife/human and wildlife/domestic animal interfaces potentially prone to pathogen spillover and to interactions negative for the welfare of wildlife (e.g., noise, hunting); and iv) the level of health intervention (e.g., control, prevention, treatment) applied to this population or interface. The various activities in which humans use (consumptively or not) wildlife species call for careful consideration about the meaning of wildlife health in heterogenous management models.

## 5 Operational framework for wildlife health

The wildlife health operational framework presented in this article combines the wildlife health approach presented in Table 1 with the wildlife categorization presented in Table 2. The aim is to better identify the health management objectives for each type of wildlife category and the domains of expertise required to address each category's health management issues (Table 3).

**Table 3:** Wildlife health framework applied to wildlife categories and their attributes using the framework adapted from Hanish et al. 2012. “Level” refers to the level of organisation of the target (i.e., individual, population or community). “Disciplines” indicates some examples of scientific disciplines needed for each component of health. “Abs.” = Absence; “Vet.” = Veterinary.

	Level	Bio-statistical theory	Disciplines	Holistic theory	Disciplines	Legal framework/national-international	Sectors/international organisations
Free-roaming wildlife	(Individual) Population Community	Natural disease dynamics	Disease Ecology	Sustainable & viable population & community	Animal Welfare Ecology Conservation and biodiversity Sc. Evolutionary biology	International conventions (e.g., CBD, CITES);	UNEP
Feral animals	Population	Abs. of public health diseases Invasive species	Disease Ecology Epidemiology	Welfare Management	Animal welfare Ecology Pest control Conservation Sc. Evolutionary biology	Various national regulations, multiple non-specific International conventions (e.g., CBD, CITES) Invasive species regulations	Vet. Services & Natural Environmental services WOAH & UNEP
Ranched wildlife	(Individual) Population	Abs. of important diseases	Veterinary Sc. Epidemiology	Welfare Economic production Management	Animal welfare Economics Tourism Game management	Species-specific regulations Livestock national regulations International conventions (e.g., CBD, CITES)	Natural Environmental services, Vet. Services WOAH & UNEP
Farmed wildlife	Population	Absence of disease	Veterinary Sc. Epidemiology	Welfare Economic production Management	Animal welfare Economics Zootechny	Species-specific regulations Livestock national regulations International conventions (e.g., CBD, CITES)	Vet. Services & Natural Environmental services WOAH & UNEP
Harvested wildlife	Individual	Absence of disease	Veterinary Sc. Vet. Public Health	Welfare Food system Management	Animal welfare Food sociology Economics	National regulation on sustainable use International conventions (e.g., CBD, CITES)	Vet. Services & Natural Environmental services WOAH & UNEP
Sport/tourism hunting wildlife	Population	Abs. of important diseases	Epidemiology Vet. public Health	Welfare Management	Animal Welfare Game management	National regulation on sustainable use International conventions (e.g., CBD, CITES)	Vet. Services & Natural Environmental services WOAH & UNEP
Wildlife pets	Individual	Absence of disease	Veterinary Sc. Public Health	Welfare (Mental Health Well-being)	Animal Welfare (Social & Psycho. Sciences)	National regulation on companion animals International conventions (e.g., CBD, CITES)	Vet. Services & Natural Environmental services WOAH & UNEP
Peri-domestic wildlife	Population (Community)	Abs. of important diseases	Disease ecology Public Health	Welfare Management	Animal Welfare Pest control	Invasive species regulation International conventions (e.g., CBD, CITES)	Vet. Services WOAH & UNEP
Zoo wildlife	Individual (Population)	Absence of disease	Veterinary Sc.	Welfare Management	Animal Welfare Zootechny (Conservation Sc.)	National & International Zoo regulations International conventions (e.g., CBD, CITES)	Vet. Services & Natural Environmental services WOAH & UNEP

The level of human interaction and management defining each wildlife category determines the range of health objectives and fields of expertise associated with the category (Table 3). The bio-statistical component defines the range of health interventions related to disease ecology and epidemiology that are needed to safeguard the health of both wildlife and the humans and livestock interacting with them. Interventions related to predicting and preventing emerging infectious diseases and pandemic prevention fall in this component [49,50]. Meanwhile, the holistic component opens avenues of health intervention that require conservation biology, ecology, management, and animal welfare expertise more than medical or epidemiological knowledge. Animal health managers and decision-makers may be surprised by this broadening of the domains of expertise needed to manage wildlife health, and will need to engage with new stakeholders equipped with the required expertise.

## 6 Discussion

The categorization of wildlife based on the type of human management and use, the resulting extent of the animal/human interface (Table 2), and the two health components presented in Table 1 provide an operational framework (Table 3) to better define health management goals for wildlife in given contexts.

The process of categorizing wildlife is relevant for two reasons. Firstly, it provides international and national health institutions, including One Health platforms, with a framework to define and control interventions (e.g., surveillance, management) that target wildlife according to the context in which they are managed or used. Secondly, it helps countries to facilitate One Health approaches to wildlife health by providing a framework to assess their country's needs in terms of expertise to manage health objectives per wildlife category, identify the most appropriate regulatory authorities, and facilitate their collaboration to achieve the health objectives sought.

The bio-statistical component of health covers two main risks. Firstly, wildlife populations can threaten the health of domestic animals and humans, and vice versa. Categorizing wildlife species that interact closely with human and/or domestic populations enables countries to target precise health objectives and implement appropriate measures. For example, the use of Prairie dogs (genus *Cynomys*) as pets in the United States has triggered plague, tularemia, and monkeypox transmission events to humans [51]. Secondly, the health of free-ranging populations in their natural habitat (and the health of ecosystems) requires a different approach, one that is more laissez-faire or oriented to reducing externalities of human actions that impact health. The bio-statistical component of health of free-roaming wildlife in its natural habitat can be challenged when a key population or the species itself is at risk of extinction. In such cases, population or even individual health surveillance and control measures may be required, especially if the survival of the population/species has been threatened by anthropogenic factors such as habitat fragmentation, inappropriate agricultural practices and other human activities polluting air, water and soil [52]. A more comprehensive approach to wildlife health, one that is fully integrated with domestic animal and human health approaches, should help countries to develop more sustainable and eco-friendly agriculture and human development practices (even if current health regulation standards usually prioritize economic-centred policies over ecologically friendly ones).

The holistic component applied to different categories of wildlife offers an opportunity to move beyond an anthropocentric focus of wildlife as being a threat to human and livestock health or a resource to use. In so doing, it opens within the One Health approach the possibility to consider wildlife health as an indicator of the quality of life and well-being of individuals, populations, and communities living in healthy (social-) ecological systems. Firstly, at the individual and population level, the recent development of the livestock animal welfare field [53] and societal concerns about livestock welfare being expressed in most developed countries are prompting, albeit slowly, the wildlife realm to reconsider the way humans interact with wild animals ([54] and associated special issue). This perspective challenges the multiple “uses” of wildlife by humans such as handling, slaughtering, research and tourism (e.g., camera trapping and wild individuals [55]). Secondly, at the population and community level, recognition of the Anthropocene, of human responsibility for the deteriorating conditions of life on Earth, opens the way for a duty of care for wildlife and ecosystems [56]. Table 3 highlights that conservation, ecosystem, and social-ecological system health experts need to be engaged and actively integrate these aspects into wildlife health. *In fine*, this holistic component of wildlife health questions the relationship between humans and Nature, highlighting the need to consider the rights of non-human animals and to engage in more disruptive thinking (e.g., convivial conservation [57]).

More practically, the holistic component also concerns the viability and resilience of populations and communities (e.g., for free-roaming populations) and the productive and economic sustainability of production systems similar to those of domestic animals (e.g., ranched wildlife). This component also addresses the quality of management regimes applied to wild populations, such as the sustainability of hunting pressure on wild populations (e.g., harvested wildlife such as wild bushmeat) or of capture rates in wild populations (e.g., wildlife trade). This component also pertains to control measures for wild populations that are considered pests (e.g., feral animals, peri-domestic wildlife). These measures aim to stop these wild populations from proliferating beyond an acceptable threshold in habitats with an extended animal/human interface (e.g., urban, peri-urban, and rural areas). Other measures are intended to reign in feral populations which are negatively impacting natural animal communities (e.g., feral cat impact on island wild bird life [58]). Applying this holistic component to wildlife pets, which are individual animals hosted in human families and sometimes considered as full sentient beings (“humanized”), opens the door for the use of mental health and well-being sciences and practices (e.g., psychology) for wild pets (as for humans).

Diverse disciplines are needed to tackle the full extent of wildlife health (Table 3). Whereas the bio-statistical component focuses on classical animal health sciences such as veterinary sciences, veterinary public health, epidemiology and disease ecology, the holistic component requires disciplines that vary between categories. Ecology and conservation sciences, which aim to preserve healthy ecosystems in which healthy wild animals live, also promote healthy free-ranging wildlife populations. Conservation sciences can also be used for zoo wildlife when zoos collaborate with species conservation programmes. Economics, population management, and zootechny are disciplines that can be applied to the production of wildlife for hunting and tourism, much like in the livestock sector.

Taking responsibility for wildlife health therefore requires a wide range of skills, knowledge, and experience. According to our framework, wildlife health cannot be the exclusive responsibility of veterinary services or of environmental bodies. Instead, it requires integration between these sectors and beyond (e.g., private sector, civil society). Unfortunately, efforts to protect wildlife from the effects of human activities, such as the spillover of pathogens from domestic animals and humans, are insufficient because currently there are no dedicated institutions or mechanisms, only ad hoc efforts responding to individual threats (e.g., endangered mountain gorilla and COVID-19 [59]). Veterinary services have a sanitary role to play as key guardians of public health and food safety. However, the responsibility for free-roaming wildlife falls partly under the relevant environmental authority which holds expertise in wildlife and environmental conservation. A recent study conducted by WOAH revealed that only 49 % of the 130 countries interviewed declared that veterinary services were in charge of wildlife health management [60]. Therefore, close collaboration between the authority responsible for wildlife and the veterinary services is essential to ensure that all aspects of wildlife health are considered. This is, however, rarely the case, as highlighted by the recognition that the environmental or ecosystem component of most One Health initiatives in eastern and southern Africa is the most neglected and non-functional [61]. When intersectoral and interdisciplinary collaboration fails, it can lead to extreme interventions such as inappropriate attempts to control diseases in wildlife populations or the treatment of wildlife, plants, parasites, and microorganisms as pests to be destroyed, which systematically fail to achieve the initial sanitary objectives [48]. The One Health concept, along with the new quadripartite international collaboration on the topic, has created an international forum to further address this integration, develop tools for integration [22], and refer to existing relevant regulations (Table 3, legal framework column).

The current lack of distinction between categories and, by extension, of a clear definition of the different wildlife populations living in a country, often leads governmental health institutions to struggle or neglect to assign responsibility for wildlife health management over several sectors. Managing wildlife health does not and should not be regulated to a single sector. Instead, it requires a One Health approach that relies on intersectoral and interdisciplinary collaboration between institutions dedicated to animal, human and environment health, as is indicated by the many disciplines displayed in Table 3. By considering the different wildlife categories present in a country and the health objectives expected for each category, a government can formulate more comprehensive and efficient national wildlife health surveillance and control strategies.

A key aspect of our framework is recognizing the interdependence of wildlife, livestock, and humans in order to protect the health of all through a holistic approach. The characterization and study of wildlife/livestock/human interfaces are therefore important [15,62], as is their surveillance and management [63]. For instance, the use of sentinel populations, both domestic (e.g., [64]) and wild (e.g., [65]), can provide an avenue to better survey and monitor these interfaces. Studying the ecology of wild populations remains crucial to better understand the dynamics of pathogens and thus better protect these populations. Disease surveillance in the wildlife trade is an important option as it provides de facto access to sentinel populations at the interface (e.g., [66]). Surveillance of the wildlife/livestock/human interface offers a more effective way to manage wildlife health than an approach focusing solely on wildlife.

This paper has highlighted the current limitations in implementing the One Health approach for wildlife management, and confirms the need to carefully define the different contexts in which wildlife is managed or used, and in which health is at stake. The step-by-step approach proposed for a given country can ensure that wildlife categories appear clearly in the national legislation to clarify the allocation of responsibilities between the different authorities/ministries involved. As there are currently few international standards and little to no regulations in place for wildlife health, this framework can provide policy makers guidance on how to align future wildlife health regulations with existing domestic animal and human health regulations. For example, farmed wildlife is often managed in a way similar to livestock, which means that livestock health standards could be applied to farmed wildlife, or at least guide the drafting of their regulations. In The Netherlands, for instance, sanitary measures used for livestock production, such as mass culling, have been applied to American minks bred in fur farms affected by COVID-19 to protect humans from a potential spillover [67].

If not hunted for meat, the risks posed by free-roaming wildlife remains very low, and are usually directly linked to invasive human practices such as agriculture or trade. However, the health risks faced by wildlife populations are rising due to their increasing exposure to pathogen spillover from domestic animals and humans, and the associated diseases can threaten their viability (e.g., PPR in wild ruminants [8] or bovine tuberculosis from cattle to buffalo [68]). Wildlife health should therefore be a key component of health systems to conserve biodiversity, protect human and domestic animal health, and as an indicator of the way the relationship between people and nature is managed. The integration of wildlife health in its broadest sense into the One Health approach would provide a more holistic One Health process.

## Author contributions

CG & AC conceptualized the manuscript, provided the basis for the methodology and wrote the initial draft. MdG-W commented on the initial draft and several others, while PC, L-MdK, SM & RSI provided comments on different drafts. CG finalized the document.

## CRediT authorship contribution statement

**C. Goulet:** Conceptualization, Supervision, Writing – original draft, Writing – review & editing. **M. de Garine-Wichatitsky:** Writing – review & editing. **P. Chardonnet:** Writing – review & editing. **L.-M. de Klerk:** Writing – review & editing. **R. Kock:** Writing – review & editing. **S. Muset:** Writing – review & editing. **R. Suu-Ire:** Writing – review & editing. **A. Caron:** Conceptualization, Methodology, Writing – original draft, Writing – review & editing.

## Declaration of competing interest

No conflict of interest.

## Data Availability

No data was used for the research described in the article.
